# A Case of Relapsing Polychondritis Initiating with Unexplained Fever

**DOI:** 10.1155/2016/9462489

**Published:** 2016-02-14

**Authors:** Kosuke Hirayama, Nozomi Iwanaga, Yasumori Izumi, Satoshi Yoshimura, Kazuhiro Kurohama, Mai Yamashita, Taichi Takahata, Ryuta Oku, Masahiro Ito, Atsushi Kawakami, Kiyoshi Migita

**Affiliations:** ^1^Department of General Internal Medicine and Rheumatology, Nagasaki Medical Center, Kubara 2-1001-1, Omura 856-8562, Japan; ^2^Department of Pathology, Nagasaki Medical Center, Kubara 2-1001-1, Omura 856-8562, Japan; ^3^Department of Ophthalmology, Nagasaki Medical Center, Kubara 2-1001-1, Omura 856-8562, Japan; ^4^Department of Otolaryngology, Nagasaki Medical Center, Kubara 2-1001-1, Omura 856-8562, Japan; ^5^Department of Rheumatology, Nagasaki University Hospital, Sakamoto 1-7-1, Nagasaki 852-8501, Japan

## Abstract

Relapsing polychondritis (RP) is a rare autoimmune disease affecting the multiple organ system. Here, we describe a case of RP initially presenting with high fever. The patient was referred to our hospital for further examination of fever of unknown origin (FUO). On admission, the patient reported dry cough in addition to fever. On physical examination, her red, swollen ears were noted, attributed on histology to inflammation with auricular perichondritis. She was diagnosed with RP and treated with oral prednisone (50 mg/day); her fever and auricular inflammation resolved. The patient no longer reported cough and body temperature returned to normal and the elevated levels of C-reactive protein (CRP) were normalized. In this case, identification of the origin of fever was a challenge because of unspecific symptoms; however, awareness of the systemic manifestations of RP may lead to the prompt diagnosis and therapeutic intervention.

## 1. Introduction

Relapsing polychondritis (RP) is a rare systemic autoimmune disease of unknown etiology that is characterized by recurrent inflammation of the cartilaginous and connective tissues [1]. The most commonly affected cartilages are those of the ears; however, multiple organ involvement, including the eyes, skin, musculoskeletal system, kidneys, cardiovascular system, and central nervous system, is not unusual [2]. Patients with RP typically present with unilateral or bilateral inflammation of the external aspects of the ears. Other frequently involved structures are the cartilaginous portions of the nose, the peripheral joints, and the tracheobronchial tree [3]. Clinically, involvement of the ears and nose is often the key to the diagnosis, because the distribution of the inflammation coincides with areas of cartilaginous tissue [4]. However, patients eventually diagnosed with RP may initially present with general, nonspecific symptoms, such as fever and malaise, which may delay the diagnosis [5]. Here, we describe a patient with RP whose initial manifestation was a high fever.

## 2. Case Report

**Figure 1:**
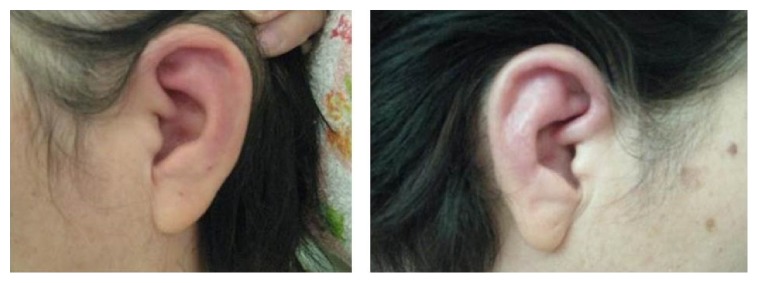
Inflammation of both ears. Obvious redness of the auricularis in this patient.

A 58-year-old female was referred to our department in January 2014 with spiking fever (>38.5°C) of 1-month duration. Examinations at local hospitals had not identified the cause of the fever and she had not responded to antibiotic therapy. At this stage, she was referred to our institution with a diagnosis of fever of unknown origin (FUO). Her history was unremarkable and she was not taking regular medications. On admission, the patient complained of chills. She had a high fever of up to 38.4°C as well as a nonproductive cough. Otological examination revealed tender and swollen pinnae (Figure 1) with characteristic sparing of the lobule. There was no abnormality of the nose or eyes.

Laboratory findings (Table 1) showed a hemoglobin level of 11.1 g/dL, a total leukocyte count of 10,700/mm^3^, and elevations of the erythrocyte sedimentation rate and C-reactive protein level. Serology tests for antinuclear antibody and autoantibodies including anti-cyclic citrullinated peptide antibody, PR3-ANCA, and MPO-ANCA were all negative.

**Figure 2:**
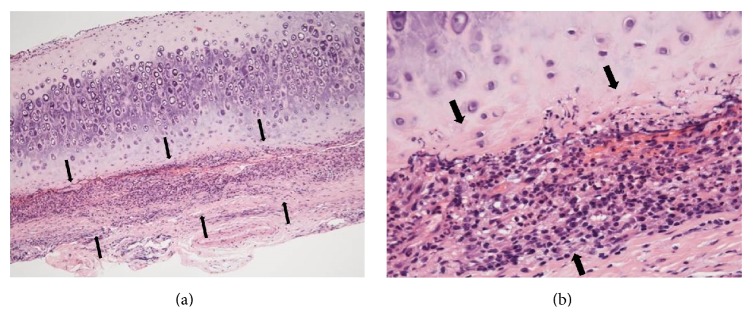
Histological findings of biopsy specimen from the left ear. Perichondritis with the mononuclear cells and polymorphonuclear leukocytes at the fibrochondral junction (hematoxylin and eosin, (a) original magnification ×50, (b) original magnification ×200). The arrows show perichondritis with the presence of mononuclear cells and polymorphonuclear leukocytes at the fibrochondral junction. Basophil's infiltration was not observed.

There was no tracheal cartilage tenderness, and chest computed tomography showed neither interstitial pneumonia nor tracheal stenosis. Neither aortic root dilatation nor aortic regurgitation was observed by echocardiography (data not shown). Although infection and hematological malignancies were excluded, redness of the bilateral auricularis suggested auricular perichondritis despite anti-type II collagen antibody negativity. Biopsy specimens were from the skin and the cartilage of the pinna for histopathological study. The histological evaluation showed cellular infiltrates of lymphocytes, neutrophils, and plasma cells, especially at the cartilage-skin interface, and a reduced number of chondrocytes in areas of cartilage destruction (Figure 2). Other clinical features of RP, such as nonerosive arthritis, ocular inflammation, and nasal chondritis, were not confirmed, whereas the patient fulfilled the McAdam-Damiani-Levine criteria for the diagnosis of RP [6, 7], according to the presence of one of McAdam's signs (auricular chondritis) with positive histological confirmation. Based on the presence of polyarthritis, the patient was diagnosed with RP. She was started on 50 mg of prednisolone daily, which led to an improvement in her symptoms. Both the fever and the auricular swelling disappeared. Tapering of her prednisolone dose in combination with the addition of 6 mg methotrexate weekly was recommended due to the suspicion of steroid-related psychiatric symptoms. Three months following her discharge from the hospital stay of 45 days, her corticosteroids were reduced to 12.5 mg daily and the methotrexate was increased to 8 mg weekly. There was no subsequent flare-up of RP.

## 3. Discussion

Relapsing polychondritis is a rare systemic disease characterized by recurrent, widespread chondritis of the auricular, nasal, and tracheal cartilages [1]. Additional clinical features include audiovestibular dysfunction, ocular inflammation, vasculitis, myocarditis, and nonerosive arthritis [8]. Although the cause remains unknown, the etiology is suspected to be an autoimmune reaction against type II collagen [9]. Established diagnostic criteria are the original McAdam's criteria, which include the presence of three or more of the following clinical features: bilateral auricular chondritis; nonerosive, seronegative inflammatory polyarthritis; nasal chondritis; ocular inflammation; respiratory tract chondritis; and cochlear and/or vestibular dysfunction [6].

A striking symptom in our patient was the spiking fever. Although there are no specific laboratory findings in RP, such as positivity for anti-type II collagen antibody, our patient fulfilled one of the McAdam criteria in addition to the typical histological findings. Patients with RP may present with various signs and symptoms that are often misdiagnosed. Among patients who present with general, unspecific signs, such as fever and progressive malaise, diagnosis and treatment may be significantly delayed [10]. The most common signs of RP are auricular inflammation (89%), nonerosive arthritis (72%), nasal chondritis (11%), and laryngotracheal disease (55%) [11]. Fever is a nonspecific sign that occurs in a wide array of disorders and its origin can be extremely difficult to determine [2]. Patients who present with RP-related fever that is not diagnosed as such may receive a diagnosis of FUO [12]. However, RP has a chronic relapsing course that can be life-threatening if there is airway involvement, and laryngotracheal involvement is a major cause of morbidity and mortality [13, 14]. Therefore, in patients whose only symptoms are prolonged fever and cough, with no pulmonary abnormalities on CT, the differential diagnosis should include RP to improve the likelihood of a timely therapeutic intervention to prevent disease progression.

Fever is often caused by the release of endogenous inflammatory cytokines in response to tissue inflammation [15]. An autoimmune reaction against type II collagen stimulates inflammatory cells, especially cytokine-producing macrophages. Glucocorticoid therapy is a fundamental component in the treatment of RP and its long-term use is recommended for most of these patients [11]. If significant organ involvement is proven, high-dose corticosteroids are often necessary. Severe disease may require treatment with immunosuppressive agents. In patients intolerant or, rarely, unresponsive to steroid therapy or in whom steroid-sparing therapy is required, immunosuppressants such as methotrexate, azathioprine, and cyclosporine play a role, particularly when there is severe respiratory or vascular involvement [16, 17]. Airway involvement by RP is considered to be a common course of morbidity and mortality [13], whereas it was reported that laryngotracheal involvement was seen less frequently in an Asian population [18]. Our patient was successfully treated with glucocorticoid and methotrexate, which were selected for their steroid-tapering effects. Associations with autoinflammatory disorders, such as familial Mediterranean fever (FMF), were reported in RP [19, 20]. In the present case, there was no relapsing periodic fever and sustained high fever was completely cured by steroid therapy; therefore, overlapping FMF in RP seems to be unlikely.

In summary, we presented the case with unexplained fever that was later determined to be an early manifestation of RP. FUO may be one of the various and nonspecific presenting features of RP. Physicians should therefore be aware of the various systemic manifestations of this disease to enable its prompt treatment.

## Figures and Tables

**Table 1:** Laboratory findings on admission.

Peripheral blood	
Red blood cells	358 × 10^4^/*μ*L
Hemoglobin	11.1 g/dL
Hematocrit	33.4%
White blood cells	10700/*μ*L
Neutrophil	80.0%
Monocyte	5.0%
Lymphocyte	15.0%
Platelet	41.7 × 10^4^/*μ*L
Blood chemistry	
Total protein	7.3 g/dL
Total bilirubin	0.5 mg/dL
Glutamic-oxaloacetic transaminase	19 IU/L (7–33)
Glutamic-pyruvic transaminase	26 IU/L (5–30)
Lactate dehydrogenase	139 IU/L (119–229)
Alkaline phosphatase	545 IU/L (80–250)
Gamma-glutamyl transpeptidase	109 IU/L (5–55)
Creatinine kinase	24 IU/L (60–160)
Total cholesterol	201 mg/dL
Blood urea nitrogen	13.5 mg/dL
Creatinine	0.5 mg/dL
Alb	3.2 g/dL
Na	138 mEq/L
K	3.9 mEq/L
Cl	101 mEq/L
Serological tests	
C-reactive protein	11.64 mg/dL (<0.30)
Erythrocyte sedimentation rate	72.0 mm/hr
Ferritin	548 ng/mL (<170)
C3	161 mg/dL (86–160)
C4	32 mg/dL (17–45)
ANA	(—) (<40)
Anti-CCP Ab	<0.6 U/mL (<4.5)
MPO-ANCA	<1.0 U/mL
RR3-ANCA	<1.0 U/mL
Type II collagen Ab	15.0 EU/mL (<25.0)
IgG	1580 mg/dL (900–2000)
MMP-3	65.1 ng/mL (<59.7)
Microbiological test	
HCV-Ab	(—)
HBsAg	(—)
CMV-antigenemia	(—)
Blood culture	(—)
*β*-D-Glucan	<3.4 pg/mL
Urinalysis	Normal

ANA: antinuclear antibody, ANCA: antineutrophil cytoplasmic antibody, CMV: cytomegalovirus, HBsAg: hepatitis B surface antigen, HCV: hepatitis C virus, MMP-3: matrix metalloproteinase-3, MPO: myeloperoxidase, RF: rheumatoid factor, and RR3: proteinase 3.
